# Lighting-from-above prior in biological motion perception

**DOI:** 10.1038/s41598-018-19851-8

**Published:** 2018-01-24

**Authors:** Leonid A. Fedorov, Tjeerd M. H. Dijkstra, Martin A. Giese

**Affiliations:** 1 Section for Computational Sensomotorics, Dept. Cognitive Neurology, CIN & HIH, UKT, University of Tübingen, Otfried-Müller Strasse 25, 72076 Tübingen, Germany; 2 Max Planck Institute for Developmental Biology, Spemannstrasse 35, 72076 Tübingen, Germany; 3 International Max Planck Research School for Cognitive and Systems Neuroscience, University of Tübingen, Spemannstrasse 38, 72076 Tübingen, Germany

## Abstract

The visual system is able to recognize body motion from impoverished stimuli. This requires combining stimulus information with visual priors. We present a new visual illusion showing that one of these priors is the assumption that bodies are typically illuminated from above. A change of illumination direction from above to below flips the perceived locomotion direction of a biological motion stimulus. Control experiments show that the underlying mechanism is different from shape-from-shading and directly combines information about body motion with a lighting-from-above prior. We further show that the illusion is critically dependent on the intrinsic luminance gradients of the most mobile parts of the moving body. We present a neural model with physiologically plausible mechanisms that accounts for the illusion and shows how the illumination prior might be encoded within the visual pathway. Our experiments demonstrate, for the first time, a direct influence of illumination priors in high-level motion vision.

## Introduction

The perception of body motion is dependent on a variety of cues, including 2D form and motion^1,2^, but also on other cues which help to disambiguate the three-dimensional structure of the body, such as disparity^3–6^. While natural body motion stimuli often specify many cues for the disambiguation of the three-dimensional body structure, it has been shown that humans effortlessly recognize three-dimensional body motion even from strongly impoverished two-dimensional stimuli^7^. This requires the combination of ambiguous stimulus information with perceptual priors that are encoded by the visual system. The exact nature of such priors for the recognition of three-dimensional body motion remains largely unknown.

We present a new perceptual illusion that implies that the perceived locomotion direction of body motion stimuli critically depends on the prior assumption that such bodies typically are illuminated from above. Such ‘lighting-from-above priors’ have been previously found for the perception of static shapes^8–17^. However, the influence of illumination direction and shading on body motion perception has never been systematically studied. Illumination from above results in the perception of the correct locomotion direction, while illumination from below can completely flip the perceived direction of locomotion. As shown by an additional control experiment, the observed illusion is not just a side-effect of classical shape-from-shading mechanisms for the perception of static shapes, and their dependence on illumination direction. Instead, it must be based on a specific previously unknown mechanism that seems to combine temporally changing intrinsic shading gradients of object surfaces (i.e. gradients that are not caused by the object boundaries) with the perceived illumination direction.

In the following, we present two experiments. Our first experiment establishes the illusion, showing that flipping the light-source position from above to below can completely change the perceived walking direction of a biological motion stimulus. In a second experiment, we isolate the visual features that critically drive this visual illusion. Our experiments motivate a computational model that accounts for the illusion, and which proposes a way how the underlying visual prior might be encoded by physiologically plausible neural mechanisms within the visual pathway.

## Results

**Figure 1:**
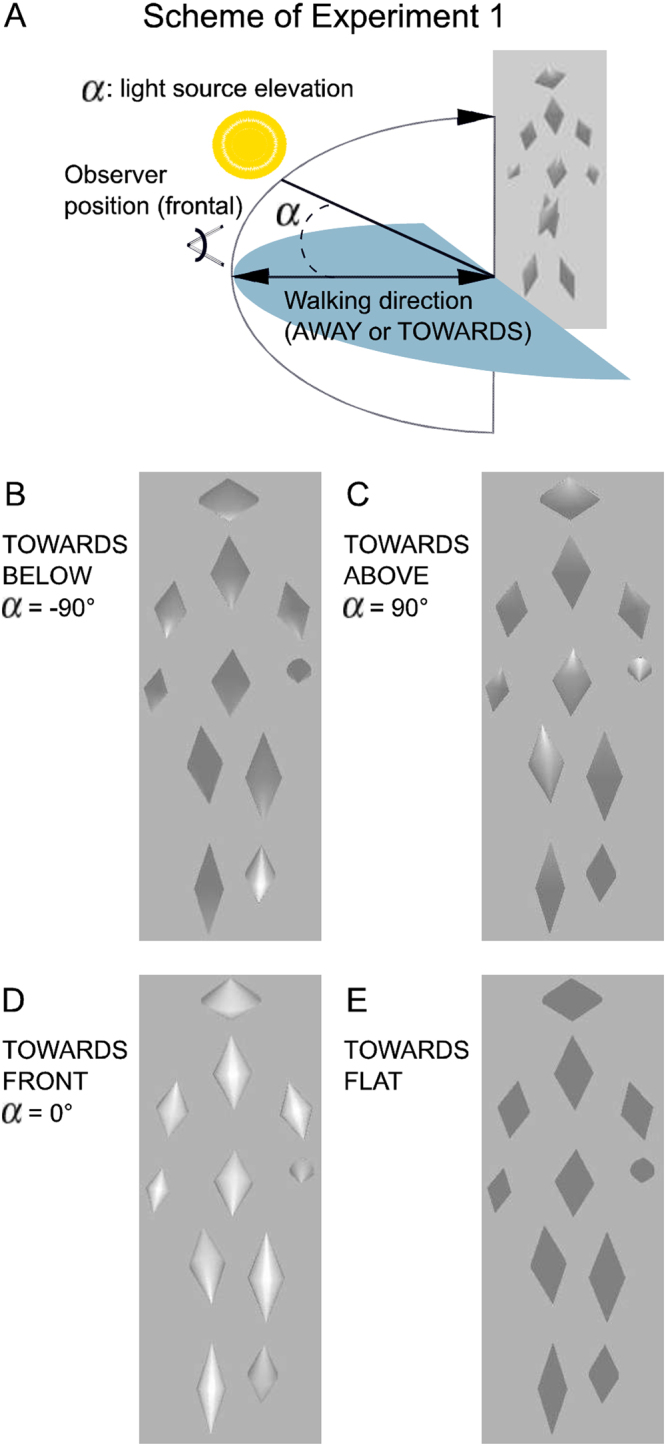
Experimental paradigm and stimuli snapshots. (**A**) Scheme of experimental setup. Participants were viewing a walker moving TOWARDS or AWAY from them. The walker consists of volumetric conic elements with a reflective grayscale surface. It was rendered assuming a light source position with a fixed elevation angle. The walker performed two gait cycles before participants were asked to report the perceived walking direction. (**B**–**D**) Characteristic snapshots of the same body configuration of the walker during the TOWARDS gait with light source positioned at different elevation angles. (**E**) Snapshot of the walker with ‘flat’ shading with uniform shading within the individual elements. Movie 1 shows these 4 walker stimuli.

**Figure 2:**
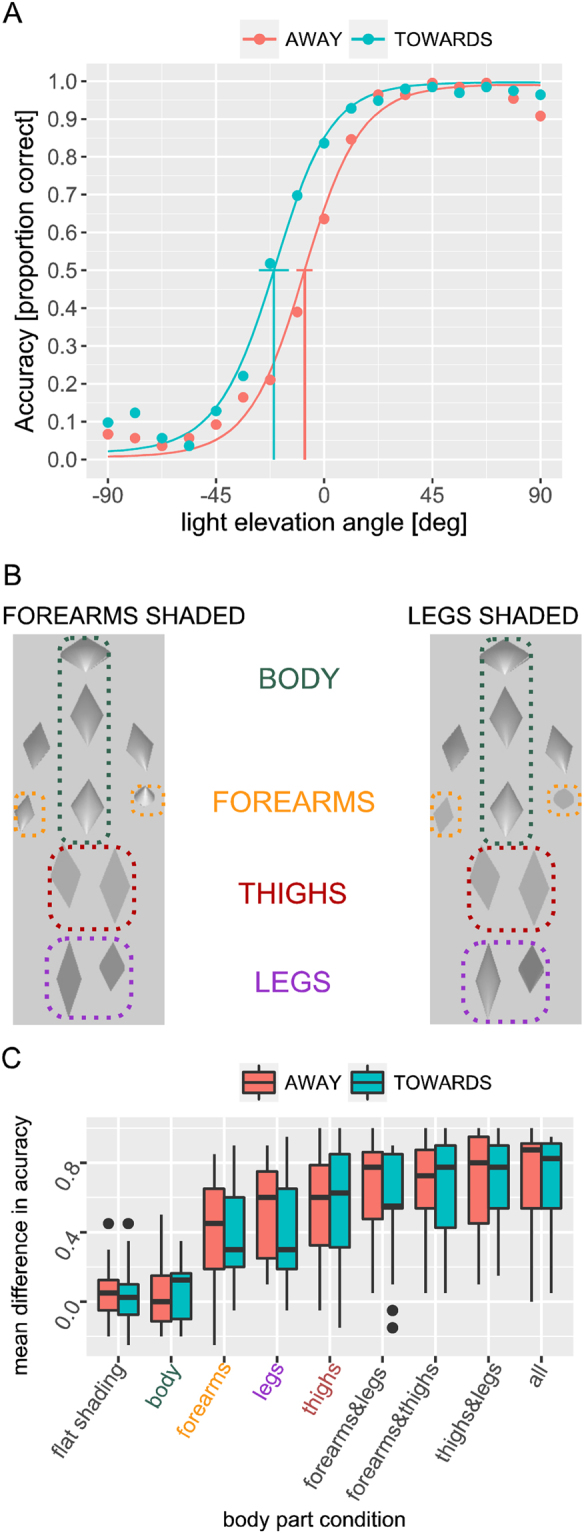
Experimental Results. (**A**) Results of Experiment 1. Accuracy of reporting the veridical walking direction as a function of the light source elevation angle α. Accuracy is defined as a probability of perceiving the true walking direction. Plotted points represent the means of the veridical binary responses per condition. The psychometric function was fitted with a generalized linear mixed effects model using cosine and sine of the light elevation angle, and walking direction as predictors (GLMM). (**B**) Snapshots from example stimuli lit from BELOW walking AWAY. Walkers illuminated from ABOVE and BELOW (light elevation angles ± 45 deg) were presented for which the gradual shading was removed from different combinations of stimulus elements. Left: ‘forearms’ condition where gradual shading was removed from the thighs and the legs. Right: ‘legs’ condition where gradual shading was removed from the thighs and the forearms. Except for the ‘flat shading’ condition the trunk and the upper arms always had gradual shading. Movie 2 shows these 2 walker stimuli. (**C**) Results of Experiment 2. Boxplot of the mean difference of the response accuracies (probabilities of correct reporting of the veridical walking direction) between stimuli illuminated from ABOVE and BELOW. This measure of the size of the illusion is shown for different combinations of elements with gradual shading. Boxes indicate the ranges of the data (middle 50% interquartile range (IQR)). Black thick lines within the boxes indicate the medians. Whiskers mark intervals of 1.5 times the IQR ranges, and the dots indicate outliers that do not fall within these intervals.

To investigate the influence of illumination direction on the perception of walking direction, we developed a novel biological motion stimulus, consisting of 11 conic volumetric elements with reflectional symmetry (Figs 1A and 2B–E). The movements of the elements were derived from motion-captured movements of a human walker (see Methods for details). It is well-known that 2D images of illuminated three-dimensional surfaces specify shading gradients that allow an estimation of the surface orientation. This estimation is also known as classical ‘shape-from-shading’ problem^18^. In this paper we investigated the influence of the such shading gradients on the perceived locomotion direction from a biological motion stimulus that consists of volumetric elements.

In Experiment 1 the elements were illuminated by a light source whose position was systematically varied (Fig. 1A). Previous work on the perception of walking from point-light stimuli has shown that direction perception can become ambiguous for particular view angles if no additional depth cues are provided^3,6^. The view of the body was chosen to minimize occlusions between different stimulus elements (see e.g. Figure 2B–E), which maximizes the ambiguity in absence of shading cues because occlusions provide relative depth information^19^. The true walking direction of the walker was either straight out of the image plane in the direction of the observer (TOWARDS) or into the image plane away from the observer (AWAY). The light source elevation angles α (Fig. 1A) varied between 90 deg (illumination exactly from above) to −90 deg (illumination exactly from below). Elevation angle α = 0 deg corresponds to an illumination directly from the side. Within a forced-choice task, participants responded whether the walker was perceived as walking ‘towards’ them or ‘away’ from them.

Participants always reported perceiving a walking human character. Whether this character was perceived as walking towards them or away from them depended on the light source elevation angle. Figure 2A shows the accuracy of the responses (proportion of correct responses where the reported direction matched the true walking direction of the walker) averaged over 13 observers (represented as points in Fig. 2A for illustration). Individual accuracies are reported in Fig. S1 in the Supplemental Information (SI). Illumination from above (α > 20 deg) results typically in correct perception of the veridical walking direction, while Illumination from below (α < −40 deg) results in an illusion: the perception of walking opposite to the veridical direction. In an intermediate regime of elevation angles (about −40 to +20 deg) the stimulus was multi-stable and the percept alternated between the two veridical walking directions. Within individual trials, observers never reported switches during the stimulus presentation. Figure S1 (SI) shows that the illusion was present in the responses of all 13 observers.

Responses of all observers were fitted with a logistic mixed-effects model (see Methods) with the cosine and sine of light angle and veridical motion direction both as fixed and random effects. This analysis uncovers systematic effects for all observers while still allowing for individual differences. The resulting fixed effects curves are plotted in Fig. 2A, one for AWAY and one for TOWARDS and the random effect ones are plotted in Fig. S1. The fits showed a highly significant effect of light angle (p < 10^−16^) on the perceived walking direction for both true walking directions (AWAY and TOWARDS). In addition, our analysis revealed a significant small effect of walking direction (p < 0.05). The small significant effect of walking direction is consistent with a bias that favors perception of walking ‘towards’ the observer, which has been also observed in previous studies with point-light-walkers^20^. A further analysis of the condition with frontal lighting (cf. Fig. S2) reveals that the veridical walking directions can be perceived with a performance above chance level even for this condition. This might be explained by the presence of subtle temporally changing shading variations within the stimulus elements even for this illumination condition.

Summarizing, the results of Experiment 1 show that the perception of body motion is influenced by a ‘lighting-from-above prior’. For illumination from above the walking direction is correctly perceived from the 2D stimulus and identical with the veridical locomotion direction of the 3D stimulus. Illumination from below, however, results in a misperception where the walker is perceived as walking in the opposite direction of the veridical locomotion.

Our stimuli were designed in a way that minimizes occlusions between different stimulus elements. Occlusion is a strong relative depth cue, which also could disambiguate the three-dimensional structure of our biological motion stimuli. Since this cue was minimized, this leaves mainly shading variations within the elements (intrinsic shading gradients) as possible depth cue. This motivated us to investigate what happens when we eliminate all intrinsic gradual shading cues from our stimuli. For this purpose, we replaced the luminance values of all pixels belonging to an element by the average luminance, averaging over all pixels that form the element and over all frames of a gait cycle. The resulting stimulus elements have a ‘flat’ shading profile that was constant over time within the elements (Fig. 1E). Note that this flat shading stimulus is different from the stimulus with frontal lighting, which still contains small changing shading gradients within the moving stimulus elements that change their orientation relative to the light source (Fig. 1D). We embedded trials with flat shading in the stimulus trials with gradual shading (as presented before in Fig. 1B–D). Consistent with our expectation, stimuli with flat shading were perceptually ambiguous and sometimes perceived as walking towards and sometimes as away from the observer. To test if there was any information about walker direction used by the observers, we fitted a logistic mixed-effects model using only an intercept as predictor to the data from trials with flat shading. We found a small but significant (p < 0.05) negative deviation of the response accuracy from chance level, i.e. observers performed worse than chance. Thus, observers made no use of the remaining information about walking direction in the flat shaded stimuli. If anything, the remaining information resulted in the perception of the wrong walking direction. Further statistical analysis results on this stimulus class is presented in the SI and Fig. S3. This result implies that the information about the walking direction is largely carried by the gradual shading within the stimulus elements, while variations of the movement kinematics and shape variations of the boundaries of the stimulus elements are apparently not exploited by the visual system even though they also contain information about the walking direction.

The new illusion was further investigated in Experiment 2, in which we varied the amount of shading information provided by the individual stimulus elements. As illustrated in Fig. 2B, we removed the shading from combinations of elements (e.g. the ones forming the forearms or the legs). In total, we used a set of nine stimuli, ranging from the original fully shaded stimulus to a stimulus with flat shading within all stimulus elements (Fig. 1E). Specifically, we removed the gradual shading from the elements forming the head, torso, the forearms and upper arms, the thighs and the lower legs. The veridical motion of the walker was again either AWAY or TOWARDS the observer. As in the first experiment, participants responded within a forced-choice task whether they perceived the stimulus as walking towards or away from them. For this experiment, we used only two light source directions (α = −45 deg and α = 45 deg), which showed large differences in accuracy between illumination from above and from below for fully shaded stimuli in Experiment 1.

The results from the second experiment are shown in Fig. 2C, separately for the stimuli with veridical motion AWAY from and TOWARDS the observer. The figure shows the mean difference in accuracy between the lighting from above and below conditions for stimuli with gradual shading in different combinations of stimulus elements (see Fig. S4 for per-observer averages). The differences were averaged over repetitions, where the colored bars indicate the ranges of the means across the different participants. The mean difference in accuracy between the two illumination conditions characterizes the size of the illusion. Consistent with our expectation, the size of the illusion increases with the fraction of elements with gradual shading. Consistent with the findings in the first experiment, for the condition with gradual shading of all elements (‘all’) we observe a large difference in accuracy (0.85), which is close to the one found in Experiment 1 (0.88). For all conditions with gradual shading of stimulus elements in the forearms, the legs, the thighs or combinations of them the difference in accuracy deviated significantly from zero (one-sample t test, p < 10^−4^ for both AWAY and TOWARDS). Contrasting with this result, stimuli without any gradual shading within the elements (labeled “none”) and the ones with gradual shading of the elements that form the head, torso and the upper arms (labeled “body”) show mean differences in accuracy that do not deviate significantly from zero (one-sample t test, p > 0.15). This indicates the absence of the illusion for those stimuli. A more detailed statistical analysis using a linear mixed-effects regression^21^ is presented in the SI. This analysis confirms that the head, torso and upper arm elements do not contribute to the illusion, while forearm, thigh and lower leg elements induce a significant illusion. In addition, the analysis shows that of the three groups of moving elements, the forearms are least effective in inducing the illusion, followed by the (lower) legs, whereas the thighs were most effective in inducing the illusory effect. From this we conclude that the gradual shading cues from the mobile elements of the walker are critical for the illusion, since in our stimulus the elements representing the head, the torso and the upper arms do not show much motion.

To further support our conclusion that the illusion is driven by the intrinsic shading gradients in the mobile stimulus elements, we developed a computational neural model that recognizes body motion by an analysis of luminance gradients. The model is based on a hierarchical neural architecture and is compatible with facts known about the visual pathway. The model learns a perceptual prior from training data that contains only stimuli that are illuminated from above. We demonstrate that this model reproduces the illusion shown in Experiment 1 and that it also reproduces qualitatively the results about the most informative features from Experiment 2.

**Figure 3:**
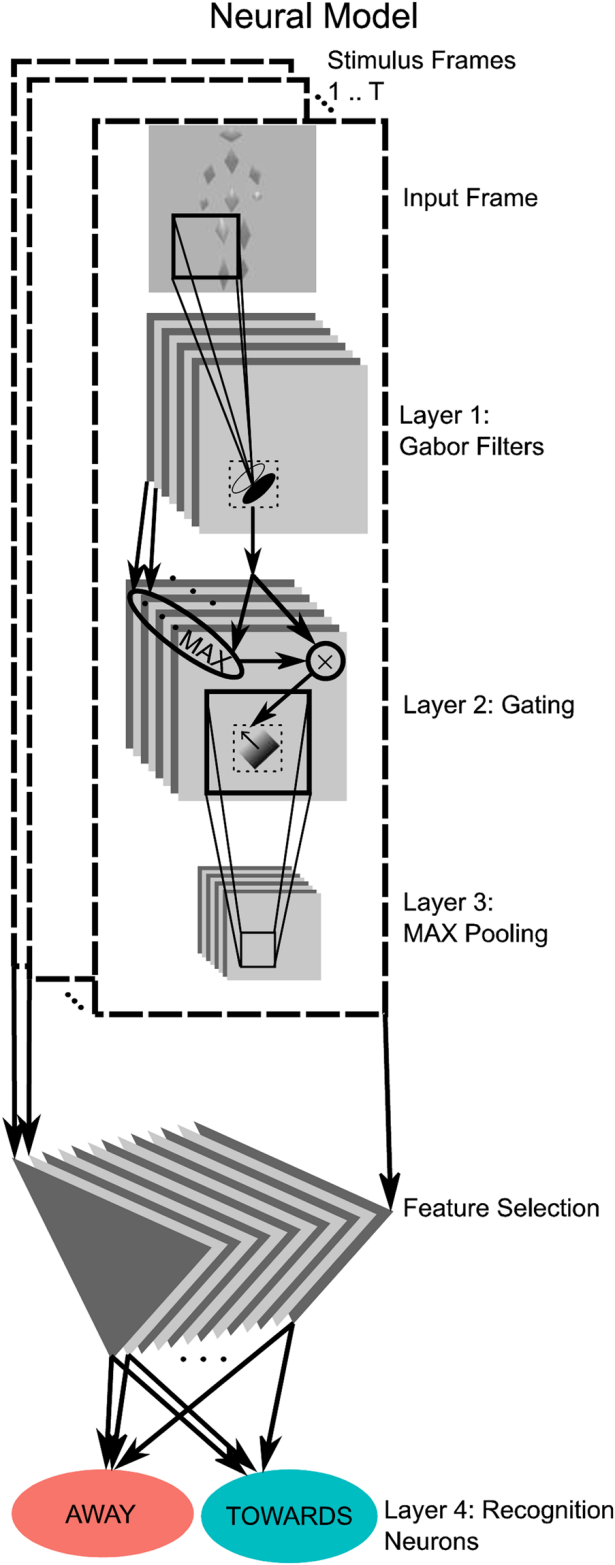
Neural model. The model consists of four neural layers: (1) uneven Gabor filters that are sensitive to shading gradients, (2) a gating stage that suppresses the strong contrast edges on the boundary of the silhouette of the walker; (3) partly position-invariant neurons that detect the strengths and direction of luminance gradients within the individual parts of the moving Figure; (4) a recognition level that processes selected features transmitted from the previous level. This level is composed from two Gaussian radial basis functions units that are trained to approximate the statistics of training patterns, which all have been illuminated from above. (See text and SI for further details.).

The model is illustrated in Fig. 3. It is formed by a hierarchy of four layers that consist of neural detectors. The first layer consists of Gabor filters, modeling V1 simple cells, where the uneven Gabor filters estimate local luminance gradients. The second layer performs nonlinear gating to suppress the strong gradients on the boundaries of the stimulus elements. Since typically the background contrast is different from the one of the stimulus element this creates strong contrast edges, which without suppression would dominate in the higher levels of the hierarchy. The gating operation suppresses the responses of the detectors to these contrast edges. The third layer pools these gated filter outputs over limited spatial regions using a maximum operation, resulting in detector responses with increased position invariance^22,23^. To determine the connections to the fourth layer we applied a feature selection algorithm, which selects only those receptive fields in layer 3 whose responses vary significantly over the training set. A further reduction of the dimensionality of the feature space is accomplished by Principal Component Analysis (PCA). The resulting reduced feature vectors provide input to the highest layer that consists of two Gaussian radial basis function units, which model a two-component Gaussian mixture distribution mixture (one component encoding AWAY and the other TOWARDS walking). The parameters of this distribution were learned in an unsupervised manner from stimuli from both veridical walking directions that were illuminated from above (α = 78.75 deg). Our model thus assumes that the visual system is trained with typical stimuli, which are illuminated from above, implementing a learned perceptual prior. The ‘perceptual response’ of the model was then determined by the radial basis function unit with the largest response, where it can be shown that this decision rule implements a Bayesian classifier. (See SI for further details).

**Figure 4:**
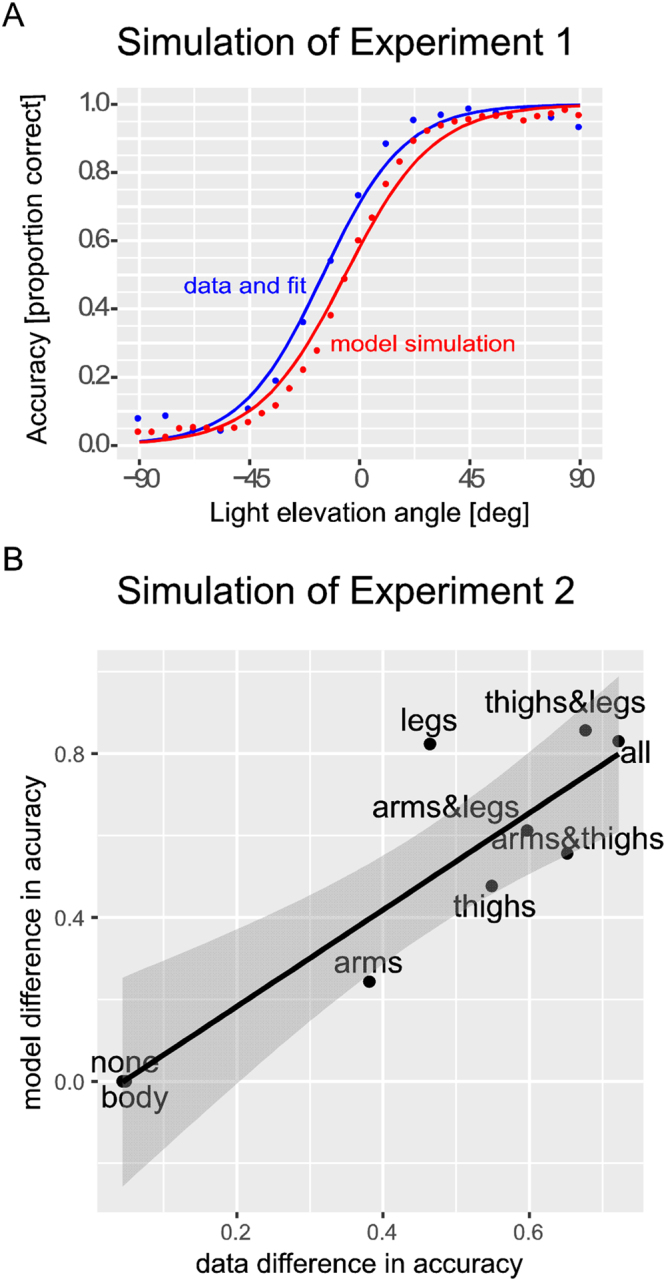
Simulation of experiments by the model. (**A**) Simulation of Experiment 1. The model reproduces the illusory effect, closely approximating the functional form obtained from the experimental data. Psychometric functions are averaged over patterns with the veridical walking directions AWAY and TOWARDS. (**B**) Simulation of Experiment 2. Separately for each stimulus type in Experiment 2, the correlation plot shows the mean differences of accuracies between illumination from above and below (cf. Figure 2C), as computed from the experimental data and the model predictions. The correlation between both measures is high (adjusted R^2^ = 0.7695) and significant (p < 0.01).

When the model was tested with the stimuli from Experiment 1 it reproduced the experimentally observed illusion. This is illustrated in Fig. 4A that shows the probability to classify the veridical walking direction of the walking stimuli, which were illuminated from the same directions as in the experiment. Like the human participants, the model misclassifies the walking direction for stimuli that are illuminated from below. The likelihood of correct classification increases as a function of the elevation angle of the light source, consistent with the results from Experiment 1, where in our analysis we averaged the responses of the AWAY and TOWARDS conditions. (See SI for details).

We also tested the model with the stimulus variants from Experiment 2. Like in Experiment 2, the size of the illusion increases with the number of gradually shaded elements of the mobile body parts, while shading of the head and torso is not providing reliable information for the classification of the walking direction. The stimulus without gradual shading cues results in completely ambiguous responses with equal probability of both classification results. The model largely reproduces the relative importance of the individual elements for the illusion size. This is shown in Fig. 4B that shows the mean differences in accuracy (that quantifies the strength of the illusion) from the experiment and the one derived from the model. The two measures are significantly correlated (R^2^ = 0.8, p < 0.001). This result further supports the hypothesis that the illusion is based on an analysis of intrinsic luminance gradients of body motion stimuli. The higher illusion size for stimuli with shaded legs might result from the fact that during the observation of body movement humans tend to attend the body center^24^ while the model treats all body parts equally and does not account for such attentional biases.

## Discussion

We presented a new psychophysical illusion that provides evidence that the perception of body motion is influenced by a lighting-from-above prior. Using a novel biological motion stimulus that consists of moving volumetric elements, we showed that the perceived walking direction matches the veridical walking direction when the stimulus was illuminated from above. If the stimulus was illuminated from below, however, the walking direction was misperceived. This implies that body motion perception integrates the stimulus information with the a-priori assumption that the light source is typically is positioned above. While our first experiment established this novel psychophysical illusion, our second experiment narrowed down the relevant visual features. Critical for the illusion were the shading gradients within the most mobile stimulus elements. We could qualitatively reproduce the illusion, and its dependence on these critical features by a neural model that analyzes intrinsic shading gradients inside the moving stimulus elements. In the model the lighting-from-above prior was learned from training patterns that were illuminated from above. Such training reflects what humans might experience in the visual world during the maturing of the visual system. Without further training the model spontaneously shows the illusion, i.e. the misperception of walking direction for stimuli that are illuminated from below. In addition, the model reproduced the dependence on critical shading features as tested in Experiment 2.

Priors for illumination direction have been reported previously for other visual functions, including the perception of static shapes^10,13–15,25,26^, visual search, and reflection perception^27^. One might thus argue that our illusion does not reveal a new perceptual process, because it might be explained by the well-known dependence of static shape perception on illumination direction in individual frames. We argue against this criticism, maintaining the claim that our illusion reveals a novel and fundamentally different perceptual process that directly analyses the dynamically changing intrinsic shading information. In order to provide support for this claim, we ran an additional control experiment.

In this control experiment we presented stimuli that prevented the reconstruction of 3D limb orientation from individual frames, while maintaining approximately the temporally varying intrinsic shading gradients of the individual stimulus elements. For this purpose, we replaced the rigid conic stimulus elements by elements with a fixed circular shape. The intrinsic luminance patterns of these elements were obtained by spatial warping of the texture of the conic stimulus elements in Experiment 1 onto these circular shapes (see Fig. S5A and the SI for details). Control subjects observing these control stimuli (see Supplementary Movies 7 and 8) perceived the elements as ‘deforming rubber sheets’, and they were not able to reconstruct reliably the 3D orientation of these elements from individual frames. However, the illusory effect was retained for these stimuli (Fig. S5B). Fitting a logistic mixed effects models to the data as for Experiment 1 we obtained a significant illusory effect of walking direction (p < 0.001). This result provides strong support for the claim that the illusion reported in this paper cannot be explained by classical shape-form-shading mechanisms, by an estimation of 3D segment orientations in individual keyframes. Rather, it must be based on a special potentially body motion-specific process.

Our neural model predicts the illusion by learning the relevant shading cues from example movies with illumination from above. A thorough analysis of the similarities of the intrinsic shading features for walking in opposite directions for opposite illumination directions explains why the model, if trained only with patterns illuminated from above, explains the misperception of walking direction by the model. To our knowledge, our model is the first one that accounts for the influence of shading on body motion perception. Further extensions of the model account also for dynamical aspects of the multi-stable perception of such body motion stimuli, such as switching rates and hysteresis^28^, as well as for the integration of the intrinsic shading features with the contour cues of the body silhouette^29^.

In order to rule out that the observed psychophysical results, and specifically the illusion, can be explained by simple low-level motion perception, instead of a more sophisticated process related to biological motion, we performed an analysis of the average optic flow generated by the stimuli in Experiment 1. We varied the light source position and walking direction and for each condition computed responses of hypothetical motion-sensitive neurons representing the total motion energy in one of eight directions. While we found that these neural responses showed reliable differences betwwwn the different conditions, the pattern of these differences was incompatible with the observed psychophysical results (smoothness of response curves and their dependence on the light source position). Moreover, even the misclassification when the light source is flipped was not reproduced by this simplified model.

Since our model is based on simple mechanisms that, in principle, can be implemented with cortical neurons (filtering, pooling, gain modulation/multiplicative gating, template matching) it makes specific predictions about cell types in the proposed visual pathway. Neurons involved in the processing of body motion stimuli have been found in macaque superior temporal cortex^4,30^. In addition, our model postulates a suppression of the contour information on the boundary of the body silhouette. Such a suppression of information on figure boundaries has been proposed also in models for other visual functions^31,32^. Electrophysiological studies will be required to unravel whether the postulated mechanisms for shading analysis really approximate computations in the biological visual pathway.

## Methods

### Apparatus

Both experiments were performed on a Dell Precision computer using the MATLAB Psychophysics Toolbox version 3. Stimuli were displayed on a 24-inch BenQ XL2420-B LCD monitor with 1920 × 1080 pixels resolution and a refresh rate of 120 Hz. Stimuli were viewed from a distance of 60 cm.

### Stimuli

All stimuli were pre-rendered before an experimental session and were identical for all participants. The stimuli presented a movie of a walking figure with a resolution of 800 by 600 pixels. The walking figure itself always fit into a 250 × 600 pixel box. The walker performed two gait cycles (four steps) and then a text was displayed that asked for the perceived perceptual alternative. The response was given by pressing one of the two buttons on the keyboard. Subsequently, the movie with the next experimental condition was started. One gait cycle took about 1 second.

### Procedure

Different observers participated in Experiments 1 and 2. Before both experiments, participants were presented with two movies of walkers lit from a 78.75-degree elevation angle walking away and towards. In this instructional step, the movies were viewed continuously until the participants confirmed seeing the veridical walking direction in both cases. To make sure the participants can follow the experimental procedure, they were then presented with a short experimental block consisting of a 20% random subset of all conditions in the experiment. No feedback was given.

In both experiments, all conditions were block-wise randomly permuted. They were presented subsequently without breaks between the blocks. In case participants wanted a break, they could stop the stimulus sequence and continue after the break. The whole experimental procedure including the instruction phase lasted less than 1 hour in both cases.

Experiment 1 comprised 36 conditions (17 light source positions and the condition with flat shading, each presented for two veridical walking directions). Each condition was repeated 15 times, resulting in a total of 540 trials. Experiment 2 included 36 conditions (9 different combinations of shaded elements, two walking directions, and two different light source positions). Because we expected the effects in this experiment to be subtler, we used 20 repetitions, resulting in a total of 720 trials

### Participants

Thirteen volunteers (mean age 25) participated in Experiment 1 of which seven were females. In Experiment 2 sixteen volunteers participated of which 7 were females. All participants were naïve about the goals of the study and were compensated by 10 EURO per hour. After the experiments, they were debriefed and informed about the study.

### Motion capture

We used the processed motion capture data from the experiments of Roether and colleagues^33^. For both experiments we used a single female walker performing an emotionally neutral gait, as defined in the above reference. For the model simulation, we used motion capture data from 3 extra actors (2 male, 1 female) also performing an emotionally neutral gait.

### Consent

Psychophysical experiments were performed with informed consent of participants. All experimental procedures were approved by the ethics board of the University of Tübingen (Germany) and all experiments were performed in accordance with relevant guidelines and regulations.

### Rendering of the surface shading and light source position

The walker was composed of conic elements rendered as surfaces in MATLAB 2014b. The element sizes were adjusted manually to match the geometry of a walking human. We chose an infinite light source distance, resulting in parallel light rays of the illumination field (choosing white as ray color). In both experiments we varied the elevation angle of the light source. In Experiment 1 the elevation varied from −90 degrees to 90 degrees, in 17 equidistant steps, and in Experiment 2 we used the two elevation angles −45 and 45 degrees, which maximized the size of the illusory effect. We used the ZBuffer renderer, which allows to specify the parameters AmbientStrength, SpecularStrength, DiffusionStrength and SpecularExponent of the surface. AmbientStrength refers to the amount of light present at every point of a scene, while the other three parameters refer to the surface reflectance properties. The walking figure was rendered on a gray background. For both experiments, we use the settings: AmbientStrength = 0.5, SpecularStrength = 0.3, and SpecularExponent = 10. For Experiment 1 we chose DiffusionStrength = 0.5, and BackgroundColor = [0.75 0.75 0.75]. For Experiment 2 we chose DiffusionStrength = 0.4, and BackgroundColor = [0.8 0.8 0.8]. For all shaded surfaces we specified FaceColor = [0.99 0.99 0.99] and removed all surface edges. The Gouraud lighting algorithm was exploited to compute the pixel colors for the specified light source positions.

For the elements with ‘flat’ shading we set the FaceColor to a constant. To calculate its value, we individually rendered each shaded element and computed the average pixel brightness over a full gait cycle.

### Neural model

Space permits only a very brief summary of the model here and we refer to our previous work^29^ for a more complete description (parameters of the model are summarized in Supplementary Table 1). The stimulus set for training and testing of the model was generated from motion capture data from 4 actors (2 male and 2 female). From each actor, we generated 25 three-dimensional body models with randomly varying sizes of the conic elements. One of these models was identical with the one used to generate the stimuli for the psychophysical experiment. Each model was rendered for the two veridical walking directions (AWAY and TOWARDS), assuming 33 different light source positions with elevation angles that varied equidistantly between −90 deg to 90 deg.

The model was trained with the stimuli (both veridical walking directions) rendered with a single elevation angle of 78.75 degrees, simulating illumination from above and using the data from all 4 actors and 25 body shape models with varying shape parameters. This variability in the training set prevents overfitting of individual training stimuli and makes the recognition more robust. For Experiment 1 the model was tested with all generated stimuli (in total 6600) using all 33 light source positions. To simulate Experiment 2, the training set was the same, and the test stimuli were generated from, also, 4 different actors, using only two light source positions, but rendered with 9 experimental conditions with different combinations of elements with or without intrinsic shading gradients (Fig. 4A). In total, only ~3% of the movies were used for training the model of Experiment 1 and ~10.0% of the movies were used for training the model of Experiment 2.

### Data availability statement

All relevant data are available as supplementary information files, with captions included in the SI.

## Electronic supplementary material

Supplementary Information File

Dataset 1

Dataset 2

Dataset 3

Dataset 4

Movie 1

Movie 2

Movie 3

Movie 4

Movie 5

Movie 6

Movie 7

Movie 8

Movie 9
