# Supplementary material for: Lighting-from-above prior in biological motion perception
Source: Sci Rep. 2018 Jan 24;8:1507. doi: 10.1038/s41598-018-19851-8 (PMC5784142; doi:10.1038/s41598-018-19851-8)
Supplement: Supplementary file 1 — Supplementary Information File [file 41598_2018_19851_MOESM1_ESM.pdf]

Supplementary Information

Title: "Lighting-from-above prior in biological motion perception"

Authors: Leonid A Fedorov[1, 3], Tjeerd MH Dijkstra[1, 2] and Martin A Giese[1, 3]

Author Affiliation:

[1]Section for Computational Sensomotorics, Dept. Cognitive Neurology, CIN & HIH, UKT, University of Tübingen, Otfried-Müller Strasse 25, 72076 Tübingen, Germany

[2]Max Planck Institute for Developmental Biology, Spemannstrasse 35, 72076 Tübingen, Germany

[3]International Max Planck Research School for Cognitive and Systems Neuroscience, University of Tübingen, Spemannstrasse 38, 72076 Tübingen, Germany

Neural model

Our hierarchical neural model consists of four layers that implement a position-invariant recognition of walking direction based on the intrinsic gradual shading variations of the individual stimulus elements. The model reproduces qualitatively the illusion, as well as the dependence of the illusion size on the available shading information.

The first layer of the model is composed from uneven Gabor filters G_u that are ordered within a rectangular spatial grid. Such filters are sensitive to oriented local luminance gradients. We assume that the receptive field center of the filter is specified by the vector (x_c, y_c), and that its preferred gradient direction is given by the angle α. We assume further that the receptive field size is specified by the parameter σ, and the preferred spatial frequency by the constant k_0. With these parameters, the filter functions are defined as:

$$G_u(x, y; x_c, y_c, \alpha, \sigma) = \exp\left(-\frac{\tilde{x}^2 + \tilde{y}^2}{2\sigma^2}\right)\sin\left(2\pi k_0 \tilde{x}\right) \qquad (1)$$

where $\tilde{x} = (x - x_c)\cos\alpha + (y - y_c)\sin\alpha$ and $\tilde{y} = -(x - x_c)\sin\alpha + (y - y_c)\cos\alpha$. We used 810,000 spatial grid points for the receptive field centers and eight different angles [illegible](cf. Tab. 1). Assuming $I(x, y)$ as the gray-level input pixel image, the output signal of the filter with these parameters is given by the sum:

$$R^1(x_c, y_c, \alpha) = \sum_x \sum_y I(x, y) G_u(x, y; x_c, y_c, \alpha, \sigma) \qquad (2)$$

In our experiments the walking direction could not be reliably extracted from the stimuli with 'flat' shading. These stimuli specify strong luminance gradients on the boundaries of the stimulus elements, but no shading gradients inside of them. The strong gradients on the element boundaries dominate the responses of the filters on the first hierarchy layer. In a set of additional simulations, we found that a more robust processing of the weak luminance gradients inside the elements can be accomplished by suppressing the gradient responses on the silhouette boundaries. This suppression is accomplished by the second layer of our model, exploiting a multiplicative gating mechanism. The underlying operation can be implemented by a simple feed-forward network that multiplies a gating signal with the filter responses from the previous layer. The gating signal is computed from all filter responses of the first layer with the same receptive field center, taking the maximum over the responses with selectivity for

different direction angles: $C(x, y) = \max_{\alpha} R^1(x, y, \alpha)$. The output signals of the second layer are then given by thresholded products of the form:

$$R^2(x, y, \alpha) = \left[H(\lambda_1 - C(x, y))R^1(x, y, \alpha)\right]_+ , \tag{3}$$

with the Heaviside function $H(x) = 1$ for $x > 0$ and $H(x) = 0$ otherwise, the positive constant λ_1 and the linear rectifier function $[x]_+ = \max(x, 0)$. The resulting output signals form a population code of the luminance gradients inside the individual stimulus elements. This layer contains a total of 6,480,000 model neurons.

The third layer of the model pools the responses with same direction selectivity from the second level over limited spatial regions $U(x,y)$ using a maximum operation, proving a (spatially subsampled) set of detector responses with partial position invariance. Mathematically these responses were given by:

$$R^3(x, y, \alpha) = \max_{(x', y') \in U(x, y)} R^1(x', y', \alpha), \tag{4}$$

The responses of the 648 neural detectors on this level can be interpreted as 'mid-level features' within the visual hierarchy. The recognition of walking direction was based on the classification of the activation patterns, exploiting a subset of these mid-level feature-detector responses, including only those detectors whose response varied significantly over the training set. In order to determine this set of detectors automatically, we computed, separately for each position (x, y), a population vector from the detector responses with different direction specificity, which was given by the complex number:

$$p(x, y) = \sum_{\alpha} R^3(x, y, \alpha) \exp(i\alpha). \tag{5}$$

Exploiting circular statistics, we computed the variance of these population vectors (see (29) for details) and retained only the responses of those detectors whose circular variance over the whole training set exceeded a fixed threshold. This operation corresponds to a feature selection stage that identifies features with robust variation within the set of training patterns. Interestingly, the selected feature detectors correspond nicely to the body parts whose shading, according to Experiment 2, is most critical for the size of the illusion.

The highest level of the model (layer 4) is given by a classifier stage that is formed by two radial basis function (RBF) units that have been trained with example stimuli walking TOWARDS and AWAY, and which were illuminated from above. The underlying training set contained 100 movies of walkers that were lit with an elevation angle of 78.75 deg, where the veridical motion direction of 100 stimuli was AWAY and the one of the other 100 movies TOWARDS. The different movies were rendered using 4 different actors (2 male, 2 female) and randomly varying sizes of the body elements. This training

encodes a perceptual prior, which reflects statistical properties of stimulus patterns that are illuminated from above, and forms thus the implementation of the 'lighting-from-above prior' in our model.

In the model implementation, the basis function units were trained using a two-step algorithm, where this training was completely unsupervised without knowledge of the true class memberships (TOWARDS vs. AWAY) of the stimuli. First, the selected features were subject to a principle component analysis (PCA), over all training examples and stimulus frames, to further reduce the dimensionality of the feature vector. We retained only the first three principal components, explaining about 70% of the variance of the original feature vectors. This percentage was sufficient since the two walking directions were linearly separable in the corresponding reduced subspace. In order to test whether the chosen number of principle components influences the results critically, we also tested a model with 20 principal components, capturing about 90% of the variance, and obtained essentially the same model predictions (results not shown). Denoting by **x** the output vector of the PCA, we fitted the distribution of these vectors over the whole training data set by a mixture of two Gaussians, which correspond in the neural implementation to the RBFs. The mixture distribution was fitted using Expectation Maximization (EM). The underlying mixture density is given by:

$$f_{\mathbf{x}}(\mathbf{x}) = \sum_{n=1}^{2} \pi_n g(\mathbf{x}; \boldsymbol{\mu}_n, \sigma_n), \tag{6}$$

The EM procedure fits the mixture weights π_n (both very close to 0.5), and the means $\boldsymbol{\mu}_n$ and the covariance parameters σ_n of the Gaussian distributions, where we assume the Gaussian density functions:

$$g(\mathbf{x}; \boldsymbol{\mu}_n, \sigma_n) = (2\pi\sigma_n^2)^{-3/2} \exp\left(-\frac{|\mathbf{x} - \boldsymbol{\mu}_n|^2}{2\sigma_n^2} \right) \tag{7}$$

Interpreting the parameters $\square_n$ as the prior probabilities of the two classes 'walking TOWARDS and 'walking AWAY' and the functions $g(\mathbf{x}; \boldsymbol{\mu}_n, \sigma_n)$ as the likelihoods of the vectors **x** conditioned on the class C_n, according to the Bayes formula the posterior probability of the two classes C_n is given by the ratio:

$$p(C_n \mid \mathbf{x}) = \frac{\pi_n g(\mathbf{x}; \boldsymbol{\mu}_n, \sigma_n)}{\sum_{m=1}^{2} \pi_m g(\mathbf{x}; \boldsymbol{\mu}_m, \sigma_m)} = \frac{g(\mathbf{x}; \boldsymbol{\mu}_n, \sigma_n)}{\sum_{m=1}^{2} g(\mathbf{x}; \boldsymbol{\mu}_m, \sigma_m)}, \tag{8}$$

given that approximately $\pi_1 = \pi_2$. The posterior class probability can thus be computed by a simple normalization operation from the RBF neuron activities, and the most likely class can be found by simple winner-takes-all competition.

In order to compare the simulation results for Experiment 1 with the experimental data, we fitted the classification responses of the model with a logistic regression, and the experimental data with a logistic mixed-effects model (see section on statistical analysis of data from Experiment 1), using only the elevation angle as fixed and random effect predictor (smooth curves in Fig. 4A). Data was collapsed across the veridical walking directions since the model treats AWAY and TOWARDS walking equally and does not contain a special mechanism that can account for the walking towards bias that has been observed in biological motion vision (18). For Experiment 2, for each test stimulus, we computed the differences of the accuracies between the two light source positions (above and below) in order to quantify the size of the illusion. The similarity of the illusion sizes derived from the model with the real data for the different shading conditions was quantified by a linear regression analysis, predicting the illusion size obtained from the model with the ones in the experimental data (Fig. 4B).

Statistical analysis of data from Experiment 1

Statistical analysis was realized using R version 3.4, RStudio version 1.1.4, and lme4 version 1.1-12. We use ggplot2 version 2.2 to generate the statistical plots and multcomp version 1.4-6 for the multiple comparisons.

For the analysis of the conditions from Experiment 1 with graded shading of all elements we combined the responses of all 13 observers and fitted them with a logistic mixed-effects model (function glmer from package lme4) with cosine and sine of the light elevation angle and veridical walking direction both as fixed and random effects. In total, we fitted data points from 13 (observers) * 2 (veridical walking directions) * 17 (light angle directions) * 15 (repetitions) = 6630 data points. We did not include the interaction between the light elevation angle and veridical walking direction in the regression because this interaction was not significant. Fig. S1 shows the probability of perceiving the veridical walking direction as a function of light elevation angle in a separate panel for each observer (the panels are ordered by increasing difference in participant's points of subjective equality). The data points in each panel are jittered to minimize overlap (hence some probabilities seem smaller than zero or larger than one). Different colors indicate the two veridical walking directions, and the curves from the random effects fit are shown in the same color. The illusion is present for all observers and both veridical locomotion directions, while the fitted curves show different offsets (thresholds) that vary between participants, and partly between the two veridical locomotion directions.

A particularly interesting question is whether the condition with frontal illumination ($\alpha = 0$), which is the condition with the smallest luminance gradients within the different stimulus elements, conveys information about then veridical walking direction. The accuracy for this class of stimuli for the different observers is shown in Fig. S2. All participants show accuracies above 0.5, indicating that there is some remaining information about walking directions even for the stimuli with illumination from the side, potentially mediated by the subtle time-varying shading gradients in the stimulus elements that are present for this stimulus class. This stands in contrast with the stimuli with flat shading, that showed accuracies even below 0.5, indicating on average a perception of the wrong walking direction.

A further set of statistical analyses investigated potential biases in the perception of walking direction in favor of walking TOWARDS or AWAY. Such biases were studied for the class of stimuli of all shaded walker elements as well as for the stimuli with flat shading.

To quantify potential biases for the stimuli with gradual shading of all elements we analyzed the points of subjective equality (PSEs) of the random effects from the model fitted above (as plotted in Fig. S1). As shown in Fig. S3A, only observer 10 has a bias to perceive 'away', while eight participants have a bias to perceive walking 'towards' them. Further four observers have little to no bias (observers 9, 5, 13 and 12). Thus, the bias in perceived walking direction is different for different observers, but most have a 'towards' bias, consistent with results on biological motion stimuli in the literature (18).

We investigated also potential biases for the perception of walking direction for the stimuli with flat shading. Response accuracies are shown in Fig. S3B, separately for the AWAY and TOWARDS conditions. Observers are ordered by the sizes of the differences between the PSEs between AWAY and TOWARDS walking. Observers 10, 9, 5, 13 and 12, have similar accuracy for the AWAY and TOWARDS walkers, whereas all others are more accurate for the TOWARDS condition.

There is a striking analogy between the two panels, in that observers with a larger difference in PSE's while viewing the fully shaded stimuli also tend to have a larger difference in accuracy between AWAY and TOWARDS walkers while viewing stimuli with flat shading. To make this relationship explicit, we plotted the difference in accuracy for the stimuli with flat shading against the difference in PSE derived from the fully shaded stimuli in Fig. S3C. Testing the correlation between both variables, by an ordinary linear regression analysis, we found the slope of the regression line to be significantly different from zero ($p < 0.01$). This shows that the observer-specific bias in perceiving an ambiguous walker as walking towards the observer is similar for the two different stimulus classes: the fully shaded stimuli and the ones with flat shading.

Statistical analysis of data from Experiment 2

We used the same software for statistical analysis as for Experiment 1. The combined responses of all 16 observers were fitted with a linear mixed-effects model (function lmer from package lme4 (19)) with body part and veridical walking direction both as fixed and random effects. In total, we fitted 16 (observers) * 2 (veridical walking directions) * 9 (body part conditions) = 288 data points, each of which was defined by the difference in accuracy between 15 repetitions with lighting from above and 15 repetitions with lighting from below. The data was collected testing 9 levels of the factor body part, while only 8 levels were used in the main analysis, since the results for the levels "none" (flat shading) and "body" (gradual shading of head, torso and upper arms only) were not statistically different and hence combined. Fig. S4 shows all of these 288 means, with the panels ordered by increasing average accuracy difference. The trend of increasing mean accuracy with body part condition holds for most observers, although there are also clear inter-individual differences. Interestingly, there are only minor differences between the stimuli with different veridical walking directions.

In a linear mixed-effects regression analysis we found a significant effect of factor body part ($p < 10^{-15}$) and an insignificant effect of veridical walking direction ($p > 0.05$). Subsequently, we realized also selected pairwise comparisons with corrections for multiple testing (R package multcomp (20)). First, we first asked whether adding the forearms, the thighs or the (lower) legs increased the mean difference in accuracy. We found that adding each of these elements significantly increased (all $p < 0.001$) the illusion relative to the baseline condition with gradual shading of the head, torso and upper arms. Second, we compared which of the three additions, forearms, thighs or (lower) legs lead to a larger illusion and found no significant differences between these three conditions (all $p > 0.5$). Third, we asked if the addition of a second moving limb region to the first increased size of the illusion. In detail, we asked whether adding (lower) legs or thighs to the forearms increased the illusion and found that it did (all $p < 0.01$). Adding the forearms to the (lower) legs or thighs did not increase the illusion (all $p > 0.1$). Adding the (lower) legs to the thighs or the thighs to the (lower) legs did increase the illusion by a modest amount ($p < 0.05$). Fourth, we asked if adding a third moving limb region to any other two moving limb regions resulted in a significant increase of performance. Only adding of the thighs to the forearms and (lower) legs resulted in a significant difference ($p < 0.05$), while this was not the case for the addition of the other two moving regions ($p > 0.2$).

Rendering of stimuli for Control Experiment

In a control experiment we used a stimulus that does not provide classical shape-from-shading cues that allow the reconstruction of the three-dimensional orientation of the body segments. However, the elements of this control stimulus approximate the internal luminance profiles of the elements of the original stimuli used in Experiment 1. The purpose of this experiment was to rule out the possibility that the observed

illusion just is a consequence of a classical shape-from-shading mechanism that estimates the stimulus element orientations in space.

The walker for this control experiment was composed of elements with fixed circular shape in order to eliminate silhouette-based orientation cues. The internal luminance profiles of these circular elements were determined from the luminance patterns of the original stimuli in Experiment 1 by warping of the shading profiles of the conic elements onto these circular patches. For this purpose, each conic element was rendered separately on a black background. Using a Sobel edge detection algorithm, we extracted the coordinates of the boundary points of the elements. For each element we computed the center of mass (X_c, Y_c) and represented the boundary points (X_b, Y_b) in terms of polar coordinates relative to this center, i.e. $(X_b - X_c, Y_b - Y_c) = R(\phi) \cdot (\cos\phi, \sin\phi)$. To each boundary point on the conic elements we assigned a corresponding boundary point (X_b*, Y_b*) on the circular element according to the equation $(X_b * - X_c, Y_b * - Y_c) = R * \cdot (\cos\phi, \sin\phi)$, where the corresponding radius R* was given by the maximal radius of all boundary points of the conic element. This construction assigns to each point inside the conic element with polar coordinates $(\lambda R, \phi)$ a corresponding point $(\lambda R*, \phi)$ in the circular element with $0 \le \lambda \le 1$. This implies for the corresponding points within the circular element the coordinates $X_b* = X_c + R * \cdot \cos\phi$ and $Y_b* = Y_c + R * \cdot \sin\phi$. Exploiting these corresponding coordinate systems, we warped the luminance profiles of the original conic elements on the ones with circular shape using nearest neighbor interpolation. Fig. S5A illustrates example frames form the generated control stimuli. The circular elements look like deforming rubber sheets and do not provide a clear impression about orientation in space.

Control Experiment

In order to assess the illusion size for the control stimulus that prevents the use of shape-from-shading for the estimation of element orientations in space, we used only three light source positions with illumination from ABOVE and three from BELOW. Like in Experiments 1 and 2, we asked the participants to report the perceived walking direction. A new group of 12 observers participated in this control experiment. The experimental protocol was identical to experiment 1 and 2, except for the fact that performed 8 instead of 4 steps (4 gait cycles instead of 2) with 20 repetitions per condition.

The results were analyzed using the same procedure as in experiments 1 and 2. We combined the responses of all 12 observers and fitted them with a logistic mixed-effects model with light elevation angle and veridical walking direction both as fixed and random effects. In total, we fitted 12 (observers) * 2 (veridical walking directions) * 6 (light angle directions) * 20 (repetitions) = 2880 data points.

The data (Fig. S5B) shows a weaker, but significant effect of the light source position on the accuracy of perceived walking direction ($p=0.02$). This shows that the illusion was present even for stimuli which make a reconstruction of the three-dimensional orientation of individual limb segments impossible. However, taking the holistic body configuration and the structure of the inner luminance gradients into account, the visual system was able to extract the walking direction from such stimuli and shows the same illusion as for the stimuli with conic elements in Experiment 1. The observed illusion is thus not just a consequence of a classical shape-from-shading process that estimates the orientation of individual stimulus elements. Instead, the observed phenomenon must be based on a mechanism that integrates body shape and information about internal shading gradients, as for example the mechanism realized by the proposed model.

Supplementary Figure Captions

Fig. S1. Per-participant data from Experiment 1. Accuracy of reporting the veridical walking direction as a function of the light source elevation angle α. The participants are ordered by increasing difference between their AWAY and TOWARDS points of subjective equality.

Fig. S2. Accuracy of reporting veridical walking direction of the frontal lighting condition (α = 0) for the fully shaded walkers. The participants are ordered by increasing difference between their AWAY and TOWARDS points of subjective equality (same as Fig. S1).

Fig. S3. Towards walking bias derived from conditions with and without gradual shading. (A) Towards walking bias derived from the conditions with full gradual shading. The figure shows the points of subjective equality (PSE) per participant, estimated from the random effects component of the GLMM fitted to the data of Experiment 1, using the conditions with gradual shading of all elements. (B) Accuracy of reporting the veridical walking direction for the stimuli with flat shading. (Participants are ordered by increasing differences of the PSEs between their AWAY and TOWARDS walking.) (C) Correlation between participant accuracies for stimuli with flat shading (panel (B)) and the PSEs derived from stimuli with full graded shading (panel (A)) (adjusted $R^2 = 0.4831$; $p < 0.01$).

Fig. S4. Per-participant accuracies of reporting the veridical walking direction as a function of the body part condition of Experiment 2. Participants are ordered by increasing difference in their accuracies, averaged over body part conditions and veridical walking directions.

Fig. S5. Control Experiment. (A) Snapshots from the control experiment with left: one of the ABOVE light elevation angles and right: one of the BELOW light elevation angles, both walking AWAY. Shading of each individual element of the walker figure was stretched from original conic shape (used in experiments 1 and 2) to a circular boundary via interpolation. Supporting Information (SI) Movie 3 shows these 2 walker stimuli. (B) Results. Walkers illuminated from 3 ABOVE and 3 BELOW light angles were presented. Plotted points represent the means of the veridical binary responses per condition. The data were fitted using a generalized linear mixed effects model (GLMM).

Supplementary Movie Captions

Movie 1. One of the stimuli used for Experiment 1. The walker is walking TOWARDS and is lit below with -90 deg elevation angle.

Movie 2. One of the stimuli used for Experiment 1. The walker is walking TOWARDS and is lit above with 90 deg elevation angle.

Movie 3. One of the stimuli used for Experiment 1. The walker is walking TOWARDS and is lit in front with 0 deg elevation angle.

Movie 4. One of the stimuli used for Experiment 1. The walker is walking TOWARDS and has flat shading.

Movie 5. One of the stimuli used for Experiment 2. The walker is walking AWAY, lit BELOW and has it's thighs and legs flattened (FOREARMS condition).

Movie 6. One of the stimuli used for Experiment 2. The walker is walking AWAY, lit BELOW and has it's forearms and thighs flattened (LEGS condition).

Movie 7. One of the stimuli used for Control Experiment. The walker is walking AWAY and is above with 67.5 deg elevation angle.

Movie 8. One of the stimuli used for Control Experiment. The walker is walking AWAY and is below with -22.5 deg elevation angle.

Movie 9. Demo video to show two walking figures next to each other. Both walkers are rendered to walk AWAY (the camera is positioned looking at the back of the walking figure). The left walker has light source positioned ABOVE, and the right walker has the light source positioned BELOW. Both walkers are looped for multiple gait cycles. Try judging the walking direction of each of them separately. Most observers would judge the left one as walking AWAY (correctly), but the right one as walking TOWARDS (opposite).

Supplementary Table 1

**Table 1. Parameter values used to simulate the model with experimental stimuli.**		
**Parameter**	**Value**	**Meaning**
α	$\{0°, 45°, 90°, 135°, 180°, 225°, 270°, 315°\}$	Orientation of maximal direction sensitivity of neurons in Layer 1 modeled as an angle value of the 2D uneven Gabor filter.
σ	60	Gabor filter scale in Layer 1.
k_0	40^{-1}	Spatial filter frequency in Layer 1.
λ_1	25	Excitation threshold in Layer 2 exceeding which activates the gating feedback inhibitory mechanism for boundary suppression.
m	81	Number of partially-position invariant detectors at Layer 3, arranged in overlapping square grid with large spatial receptive field sizes.
T	30	Number of keyframes taken in every individual walker movie.
$X \times Y$	900×900	Resolution of a single keyframe.
$U(x, y)$	300×300	Size of spatial max-pooling region in Layer 3.

Supplementary Dataset Captions

Dataset 1. Collected participant data for Experiment 1, conditions with shaded walker.

Columns indicate, in order: participant ID, light elevation angle in degrees, walking direction, participant's binary accuracy in indicating the walking direction (1 if correct, 0 if not), participant's binary accuracy in indicating the walking direction (1 if correct, 0 if not) in the previous trial.

Dataset 2. Collected participant data for Experiment 1, single condition with flat walker.

Columns indicate, in order: participant ID, light elevation angle set to 'Inf' indicating no shading present, walking direction, participant's binary accuracy in indicating the walking direction (1 if correct, 0 if not), participant's binary accuracy in indicating the walking direction (1 if correct, 0 if not) in the previous trial.

Dataset 3. Collected participant data for Experiment 2.

Columns indicate, in order: participant ID, light elevation angle in degrees, walking direction, participant's binary accuracy in indicating the walking direction (1 if correct, 0 if not), body part condition name, recoded light source position (same as light elevation angle, coded ABOVE if 45, BELOW if -45), participant's perceived walking direction.

Dataset 4. Collected participant data for Control Experiment.

Columns indicate, in order: participant ID, light elevation angle in degrees, walking direction, participant's binary accuracy in indicating the walking direction (1 if correct, 0 if not).

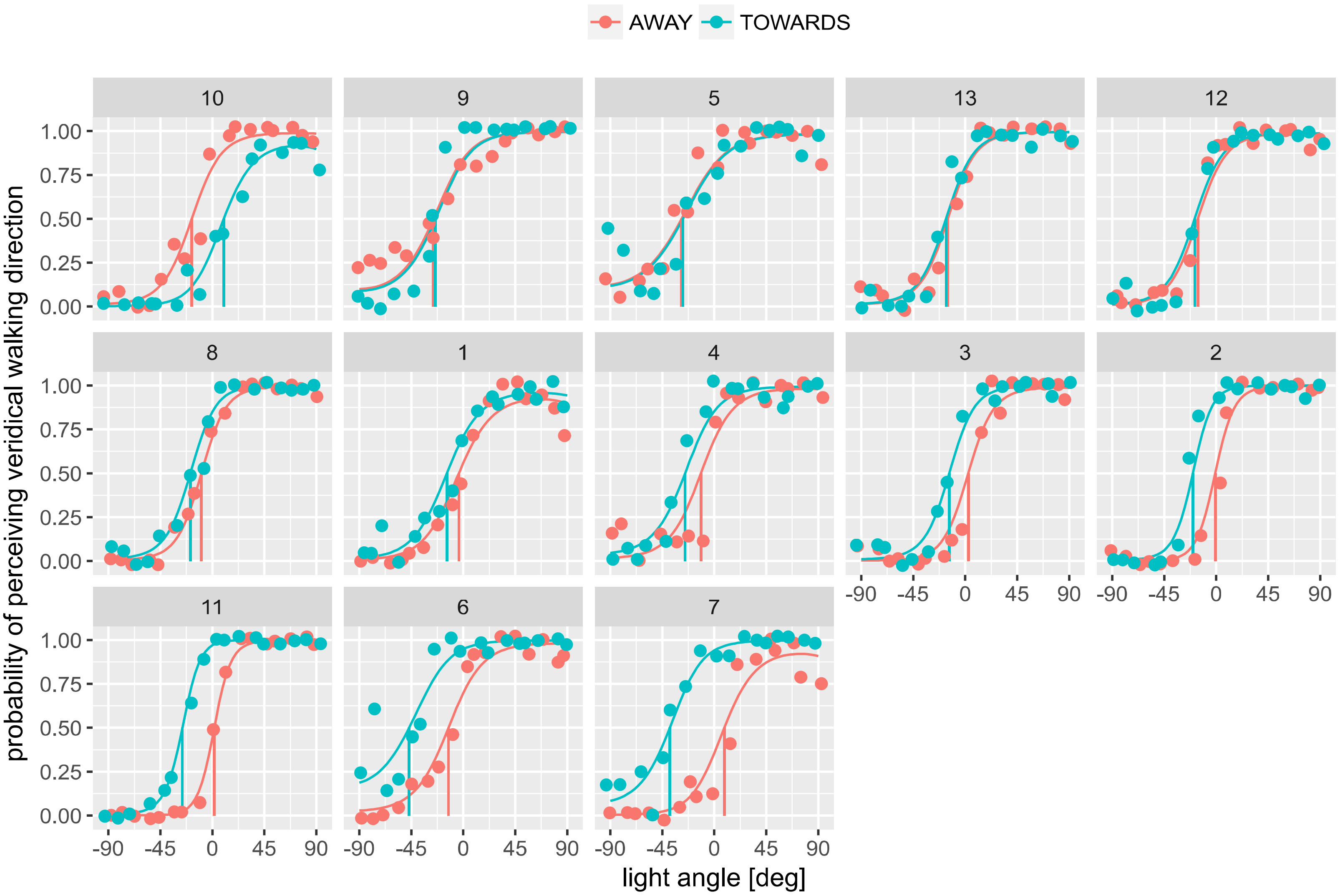
AWAY
TOWARDS
10
9
5
13
12
8
1
4
3
2
11
6
7
probability of perceiving veridical walking direction
light angle [deg]
1.00
0.75
0.50
0.25
0.00
-90
-45
0
45
90

Figure S1.

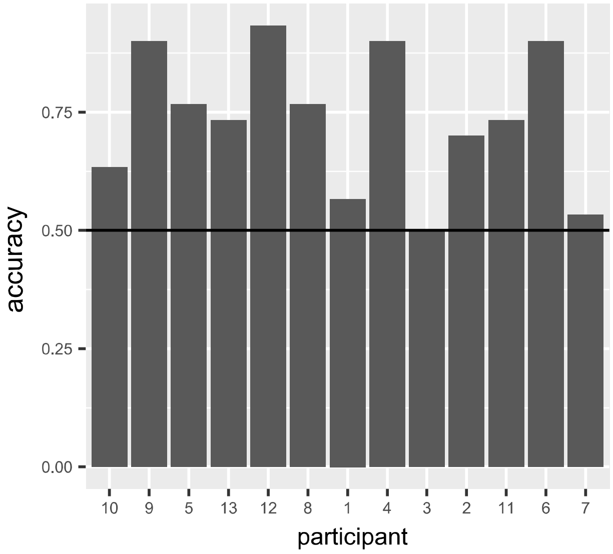

Figure S2.

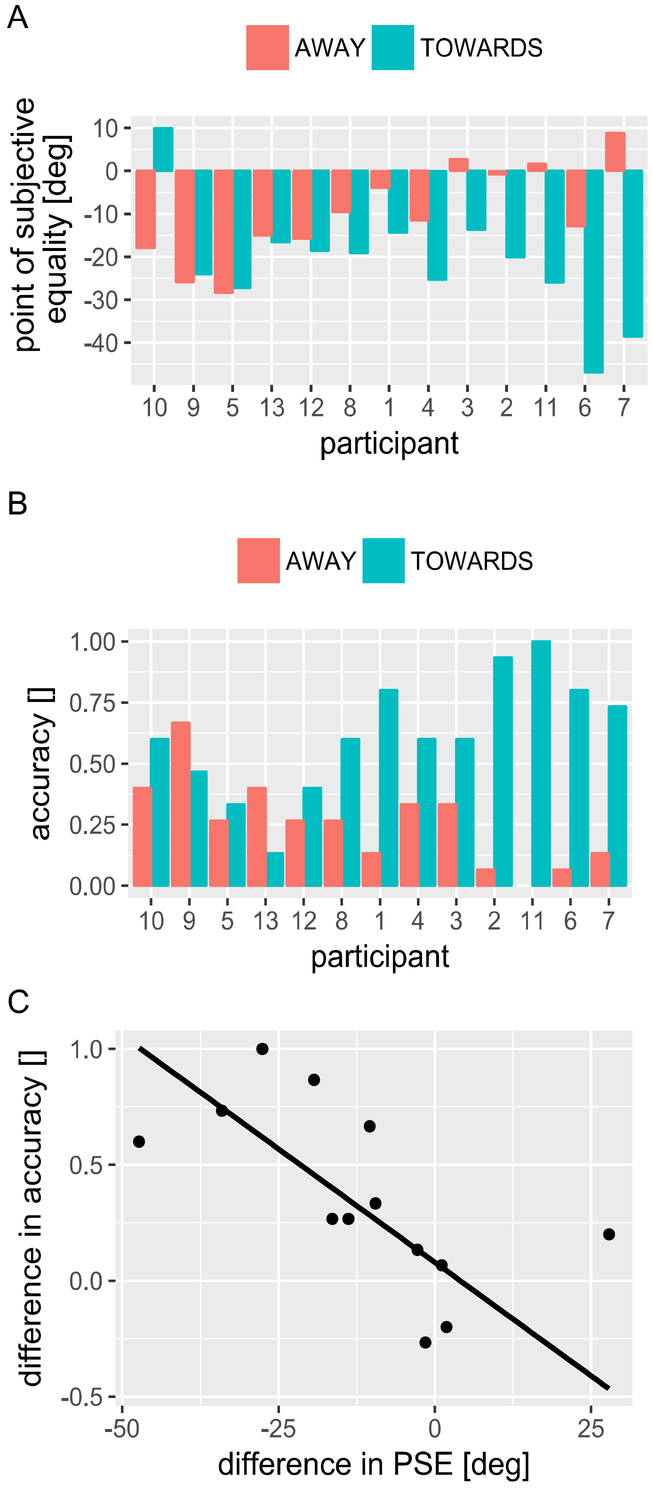

A
AWAY
TOWARDS
point of subjective equality [deg]
10
0
-10
-20
-30
-40
10 9 5 13 12 8 1 4 3 2 11 6 7
participant
B
AWAY
TOWARDS
accuracy []
1.00
0.75
0.50
0.25
0.00
10 9 5 13 12 8 1 4 3 2 11 6 7
participant
C
difference in accuracy []
1.0
0.5
0.0
-0.5
-50
-25
0
25
difference in PSE [deg]

Figure S3.

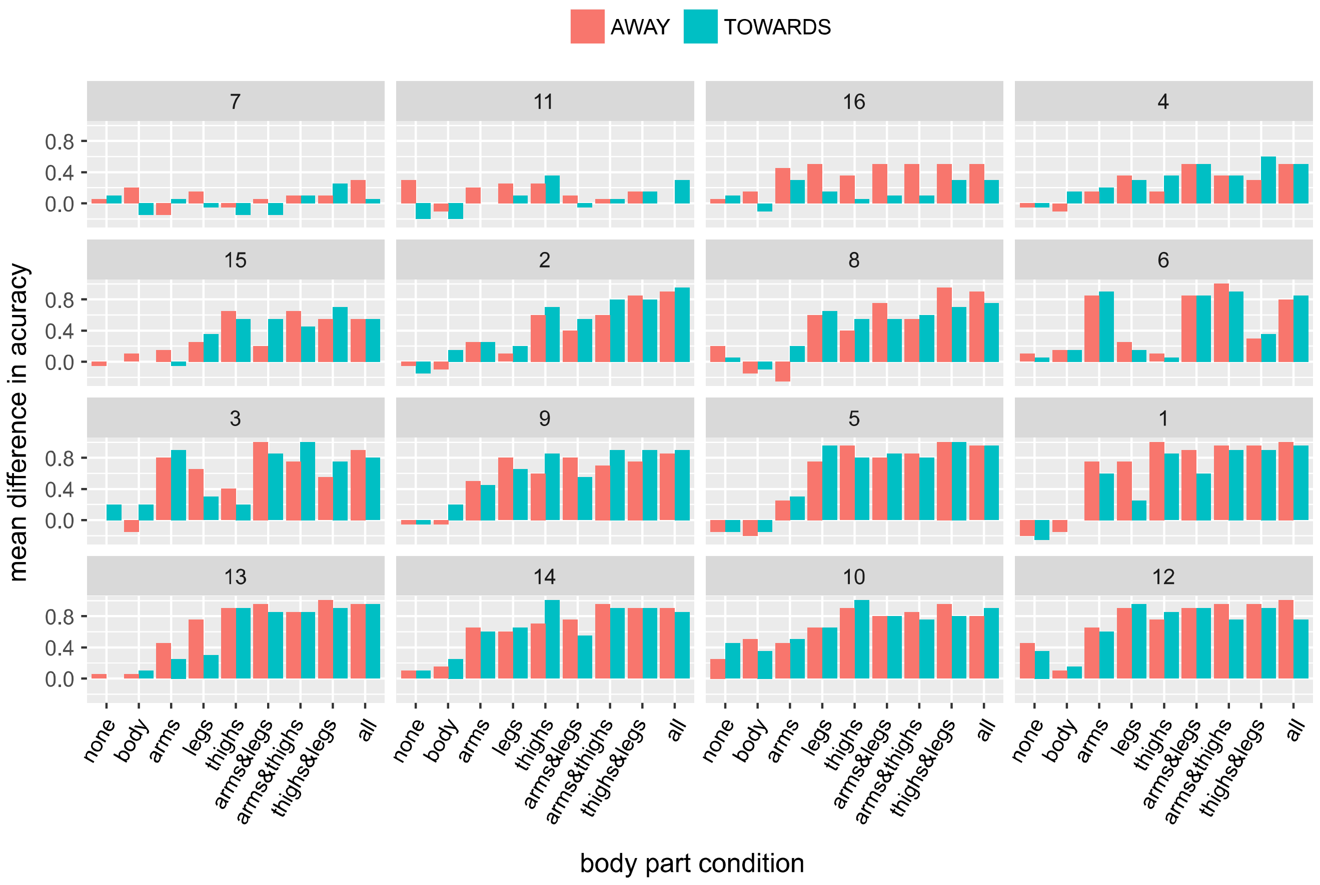

AWAY
TOWARDS
7
11
16
4
15
2
8
6
3
9
5
1
13
14
10
12
0.8
0.4
0.0
mean difference in acuracy
none
body
arms
legs
thighs
arms&legs
arms&thighs
thighs&legs
all
body part condition

Figure S4.

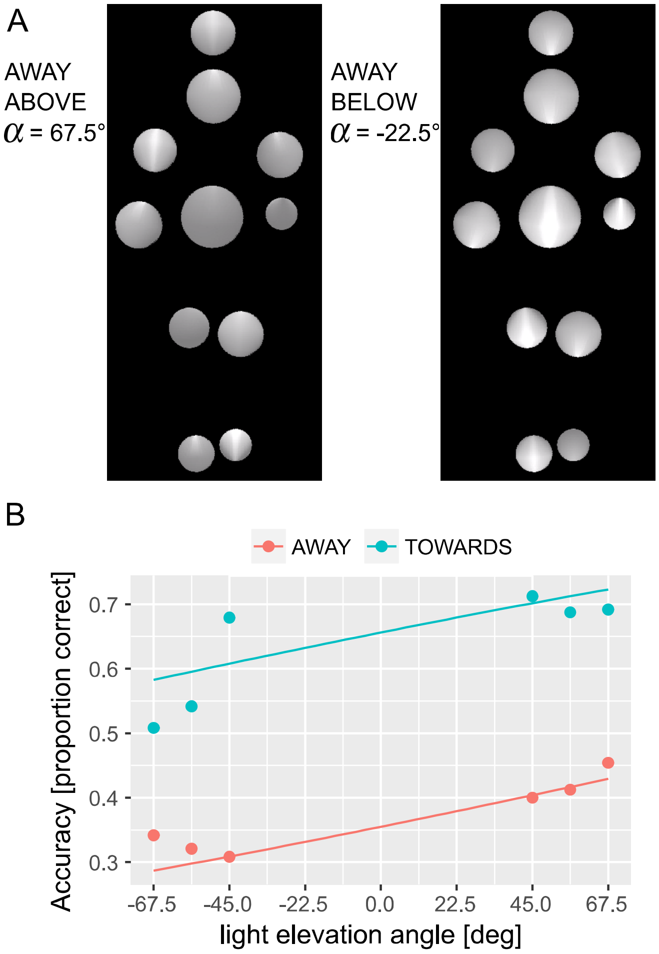
A
AWAY
ABOVE
α = 67.5°
AWAY
BELOW
α = -22.5°
B
AWAY
TOWARDS
Accuracy [proportion correct]
0.7
0.6
0.5
0.4
0.3
-67.5
-45.0
-22.5
0.0
22.5
45.0
67.5
light elevation angle [deg]

Figure S5.
